# Surface Modification of Li_3_VO_4_ with PEDOT:PSS Conductive Polymer as an Anode Material for Li-Ion Capacitors

**DOI:** 10.3390/polym15112502

**Published:** 2023-05-29

**Authors:** Shih-Chieh Hsu, Kuan-Syun Wang, Yan-Ting Lin, Jen-Hsien Huang, Nian-Jheng Wu, Jia-Lin Kang, Huei-Chu Weng, Ting-Yu Liu

**Affiliations:** 1 Department of Chemical and Materials Engineering, Tamkang University, No. 151, Yingzhuan Road, Tamsui District, New Taipei City 25137, Taiwan; roysos1@gmail.com; 2 Department of Materials Engineering, Ming Chi University of Technology, 84 Gungjuan Road, Taishan District, New Taipei City 24301, Taiwan; xian5379@me.com; 3 Institute of Nuclear Energy Research, Atomic Energy Council, 1000 Wenhua Road, Jiaan Village, Longtan District, Taoyuan City 32546, Taiwan; yantinglin@iner.gov.tw; 4 Department of Green Material Technology, Green Technology Research Institute, CPC Corporation, No.2, Zuonan Rd., Nanzi District, Kaohsiung City 81126, Taiwan; 295604@cpc.com.tw; 5 Université Paris-Saclay, CNRS, Institut des Sciences Moléculaires d’Orsay, 91405 Orsay, France; nianjheng.wu@u-psud.fr; 6 Department of Chemical and Materials Engineering, National Yunlin University of Science and Technology, Douliu 64002, Taiwan; 7 Department of Mechanical Engineering, Chung Yuan Christian University, No. 200, Chungpei Road, Chungli District, Taoyuan City 32023, Taiwan; 8 Department of Chemical Engineering and Materials Science, Yuan Ze University, Taoyuan City 32003, Taiwan

**Keywords:** Li_3_VO_4_, PEDOT:PSS, surface modification, lithium ion battery, supercapacitor

## Abstract

Li_3_VO_4_ (LVO) is a highly promising anode material for lithium-ion batteries, owing to its high capacity and stable discharge plateau. However, LVO faces a significant challenge due to its poor rate capability, which is mainly attributed to its low electronic conductivity. To enhance the kinetics of lithium ion insertion and extraction in LVO anode materials, a conductive polymer called poly(3,4-ethylenedioxythiophene):poly(styrenesulfonate) (PEDOT:PSS) is applied to coat the surface of LVO. This uniform coating of PEDOT:PSS improves the electronic conductivity of LVO, thereby enhancing the corresponding electrochemical properties of the resulting PEDOT:PSS-decorated LVO (P-LVO) half-cell. The charge/discharge curves between 0.2 and 3.0 V (vs. Li^+^/Li) indicate that the P-LVO electrode displays a capacity of 191.9 mAh/g at 8 C, while the LVO only delivers a capacity of 111.3 mAh/g at the same current density. To evaluate the practical application of P-LVO, lithium-ion capacitors (LICs) are constructed with P-LVO composite as the negative electrode and active carbon (AC) as the positive electrode. The P-LVO//AC LIC demonstrates an energy density of 107.0 Wh/kg at a power density of 125 W/kg, along with superior cycling stability and 97.4% retention after 2000 cycles. These results highlight the great potential of P-LVO for energy storage applications.

## 1. Introduction

Over the past few decades, electrochemical energy storage systems have attracted significant attention for use in electric vehicles and renewable energy sources. Among these systems, the supercapacitor (SC) and lithium-ion battery (LIB) have gained particular interest. However, SCs primarily store electrical energy at the electrode/electrolyte interface, forming an electrical double-layer, whereas LIBs store charge through the insertion/desertion of Li^+^ ions. Thus, these two types of energy storage devices suffer from low energy density and low power, respectively. Recently, a new hybrid energy storage device, the Li-ion capacitor (LIC), has been developed that successfully combines the high-power capabilities of an SC and the high-energy storage capacity of an LIB [1,2,3]. LICs typically consist of an LIB-type anode and an electrochemical double-layer capacitance (EDLC) type cathode. The LIB anode and EDLC cathode in an LIC operate through the insertion and desorption of Li+ ions and reversible adsorption and desorption of ions in a Li+ ion-containing organic electrolyte. Overall, the use of an LIB-type anode and an EDLC-type cathode in an LIC provides a balance between energy density and power density, making it a promising energy storage device for various applications. For instance, hybrid systems employing Li_4_Ti_5_O_12_ (LTO) and active carbon (AC) as negative and positive electrodes with higher energy density compared to traditional symmetric EDLCs and an excellent rate performance have been proposed [4,5]. Moreover, X. Jiao et al., fabricated an LIC composed of TiNb_2_O_7_(TNO)/graphene composite and AC electrodes, which demonstrated a much higher energy density of 86.3 W h/kg (at 237.7 W/kg) than that of AC//AC symmetric supercapacitors [6].

In recent years, LVO has emerged as a promising insertion type anode material for lithium-ion batteries (LIBs) owing to its suitable intercalation potential range of 0.5–0.8 V and a larger theoretical reversible capacity of 592 mAh/g [7], which surpasses that of LTO (175 mAh/g) [8] and TNO (387.6 mAh/g) [9]. Li_3_VO_4_ is composed of corner-sharing VO_4_ and LiO4 tetrahedral units with vacant octahedral locations that provide numerous channels for Li+ ion insertion and desorption [10] without significant volumetric changes (only 4%), ensuring superior cycling stability [11]. Nevertheless, the low electronic conductivity of LVO remains a major obstacle to achieving high rate capability, similar to the substantial drawbacks of LTO and TNO. Researchers have made extensive efforts to overcome this limitation, such as preparing composites with carbon materials [12,13], ionic doping [14,15], reducing particle size, and constructing nanomorphology [16,17,18]. Surface modification of anode or cathode materials in LIBs with conducting polymers is another efficient method to improve the electrochemical property due to their high conductivity. Among them, PEDOT:PSS is one of the most successful conducting polymers in terms of commercial and research applications. PEDOT:PSS is a blend of two polymers that form a conducting polymer composite, with PEDOT providing electrical conductivity and PSS improving the solubility and processability of the polymer. Researchers have prepared PEDOT-coated LTO, which displays a higher capacity of 141.1 mAh/g than that of pristine LTO at 10 C [19], and LTO decorated with PEDOT, which shows better electrochemical behavior with improved rate performance and cycling stability [20]. However, the effects of PEDOT on the LIB property of the LVO anode remain unclear. Therefore, in this study, we modified the LVO surface with conductive PEDOT:PSS to improve its conductivity and rate performance. Additionally, we fabricated Li-ion capacitors (LICs) based on PEDOT:PSS-modified LVO and AC to evaluate the practical application of the modified LVO.

In the present investigation, we introduce a novel anode material denoted as P-LVO, which exhibits outstanding rate capability and cycling performance. To modify the surface of LVO and enhance its conductivity, we prepared a P-LVO nanocomposite through dispersing LVO obtained by the hydrothermal synthesis in a diluted solution of PEDOT:PSS, followed by ultrasonic-assisted rotary evaporation. The improved electrochemical performance of the P-LVO anode material over that of the pristine LVO anode is attributed to the increased conductivity. Furthermore, we fabricated a Li-ion capacitor (LIC) with P-LVO//AC as the anode and cathode, respectively, and compared its properties with those of LICs based on LTO//AC and TNO//AC. The specific capacitance of P-LVO//AC LIC at 0.1 A/g was found to be 123.3 F/g, which is higher than that of the LTO//AC and TNO//AC LICs (72.9 F/g and 93.0 F/g, respectively). Our results suggest that P-LVO has promising potential for use in energy storage applications.

## 2. Experimental Section

### 2.1. Synthesis of LVO Anode Material

In this study, LVO was prepared by a hydrothermal microwave-assisted approach. In a typical procedure, NH_4_VO_3_ and LiOH with a molar ratio of 1:3 were dissolved in deionized water. Then, the mixture was transferred to a microwave-assisted hydrothermal reactor, and the mixture was heated at 180 °C for 2 h. The obtained product was purified by washing with deionized water several times and finally dried in an oven for 24 h.

### 2.2. Preparation of the PEDOT:PSS Modified LVO (P-LVO)

Firstly, 5 g PEDOT:PSS aqueous solution was diluted with 45 g deionized water. Then, 1 g LVO was added and dispersed in the diluted PEDOT:PSS solution by a probe-type sonicator for 30 min. Finally, the suspension was dried by rotary evaporation to obtain the P-LVO composite.

### 2.3. Material Characterizations

X-ray powder diffraction (XRD, Philips X’Pert/MPD instrument, El Dorado County, CA, USA) is used to study the crystal structure of the as-prepared LVO. Fourier transform infrared (FTIR, PerkinElmer, Boston, MS, USA) spectroscopy and Raman spectrum (HR800, HORIBA, Kyoto, Japan) are used to investigate the vibrational modes in LVO. Scanning electron microscopy (SEM, JEOL JSM-6701F, Tokyo, Japan) and Transmission electron microscopy (TEM, JEOL 2010, Tokyo, Japan) are performed to image the surface of the LVO and P-LVO at high magnification. The weight loss of P-LVO at elevated temperature is recorded by thermal gravimetric analysis (TGA, PerkinElmer, Boston, MS, USA). The chemical composition and electronic structure of LVO and P-LVO are analyzed by X-ray photoelectron spectroscopy (XPS, ULVAC-PHI, Chigasaki, Japan). Details of the electrochemical characterizations are provided in the Supporting Information.

## 3. Results and Discussion

**Figure 1:**
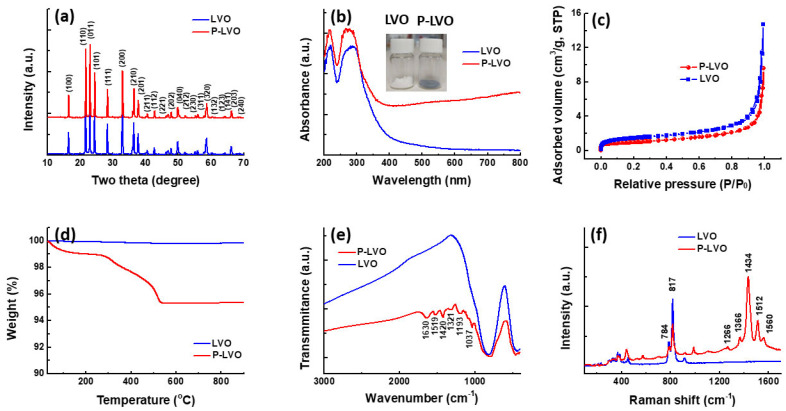
The characterization of the pristine and modified LVO. (**a**) XRD pattern; (**b**) UV-vis diffuse reflectance spectrum (the inset shows the image of pristine LVO and P-LVO); (**c**) nitrogen adsorption/desorption isotherms; (**d**) TGA analysis; (**e**) FTIR spectrum; and (**f**) Raman spectrum of the pristine LVO and P-LVO.

The crystal structure of the as-prepared LVO was analyzed using X-ray diffraction (XRD). As depicted in Figure 1a, all the XRD peaks of the samples were identified as Li_3_VO_4_ with an orthorhombic structure (JCPDS No. 38-1247), and no impurities were detected. The XRD pattern of the P-LVO remained almost unchanged compared to the pristine sample, indicating that the coating process did not affect the crystal structure of LVO. The UV-Vis spectra of LVO and P-LVO are shown in Figure 1b. The absorption edge of LVO was observed at 388 nm, corresponding to a band gap of approximately 3.19 eV. Upon coating with PEDOT:PSS, the optical absorption of P-LVO increased significantly in the visible light region, and the color of LVO changed from white to light blue (P-LVO), as shown in the inset. The nitrogen adsorption–desorption isotherms of the LVO samples are displayed in Figure 1c. Both pure LVO and P-LVO exhibited a type III isotherm without a hysteresis loop, suggesting a typical nonporous structure [21]. Moreover, the P-LVO reveals a slightly lower surface area (3.54 m^2^/g) than that of LVO (5.54 m^2^/g). To further examine the PEDOT:PSS content in the P-LVO composite, a TGA analysis was carried out, as shown in Figure 1d. It can be seen that the pure LVO exhibits great thermal stability without obvious weight loss after heating to 900 °C. In contrast, the P-LVO shows a total weight loss of 4.67% over the temperature range from 30 °C to 900 °C due to the thermal decomposition of PEDOT:PSS. The results clearly confirm that the content of PEDOT:PSS in P-LVO is 4.67%, which is consistent with the feeding ratio in the coating process. The surface functional groups of LVO and P-LVO were identified by the FTIR spectrum. As shown in Figure 1e, the two strong characteristic peaks located at 830 and 433 cm^−1^ observed in pristine LVO and P-LVO are assigned to the symmetric stretching of V−O and symmetric stretching of V−O−V, respectively [22]. For the surface-modified LVO, the peaks at 1630 and 1519 cm^−1^ are due to the asymmetric stretching mode of C=C [23,24]. The peaks appearing at 1420, 1321, 1193, and 1037 cm^−1^ are mainly attributed to the inter-ring stretching mode of C−C and C−O−C bending vibration in the ethylenedioxy group [23,25]. Raman spectra, as shown in Figure 1f, further confirm that PEDOT:PSS was presented in the P-LVO composite. The Raman bands between 250 and 500 cm^−1^, 750 and 950 cm^−1^ are attributed to the characteristic peak of the LVO, which are in good agreement with the previous report [22,26]. The Raman bands of LVO at 818 and 785 cm^−1^ are attributed to symmetric and asymmetric stretching of VO_4_^3−^ and the band at 326 cm^−1^ is generated by the bending vibration of VO_4_^3−^. Compared with the pristine sample, P-LVO shows additional Raman bands between 1200 and 1600 cm^−1^, which belong to the characteristic bands of PEDOT:PSS [27]. These results verify that the PEDOT:PSS was successfully modified on the LVO surface.

**Figure 2:**
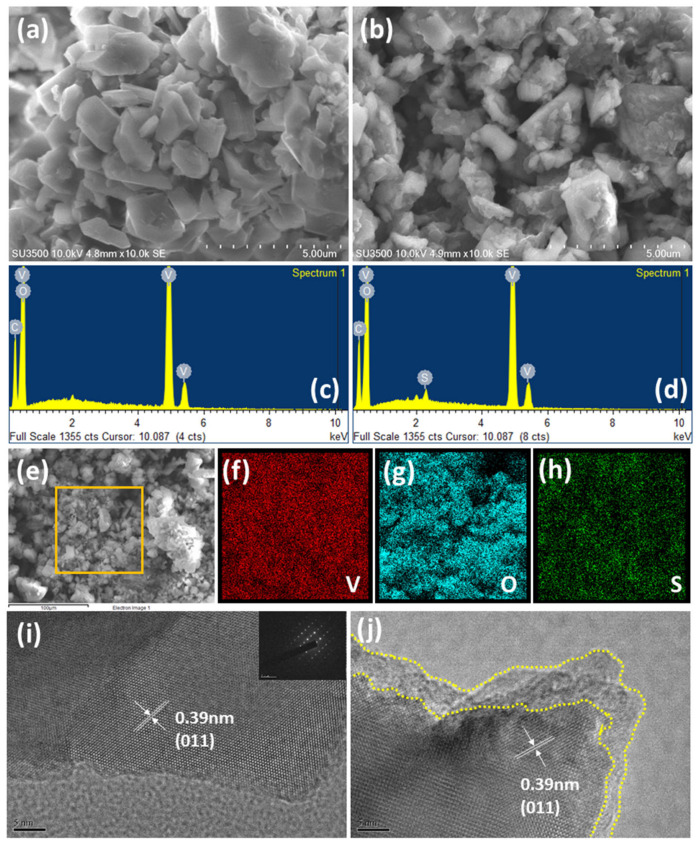
The morphological investigation of the P-LVO. The SEM images of (**a**) LVO and (**b**) P-LVO; the EDS spectra of (**c**) LVO and (**d**) P-LVO; (**e**) representative SEM image with the associated EDS mapping of the P-LVO (yellow square); (**f**–**h**) EDS mapping of (**f**) V, (**g**) O, and (**h**) S elements in the P-LVO powder; and the high-resolution TEM images of (**i**) LVO and (**j**) P-LVO.

The morphology of the two samples was investigated using SEM and TEM. The SEM images of pristine LVO and P-LVO are shown in Figure 2a,b, respectively. Both samples exhibit secondary agglomerates comprised of irregular primary particles with a well-crystallized structure and a particle size distribution (PSD) ranging between 0.5 and 2.7 μm, indicating that the surface modification process does not alter the crystalline structure or PSD of LVO. Although the surfaces of pristine LVO are smooth, some debris can be seen on the P-LVO, forming a rougher surface. The EDS spectrum of P-LVO (Figure 2d) reveals an additional S signal compared to pristine LVO (Figure 2c), indicating the presence of PEDOT:PSS in P-LVO. The marked region in Figure 2e was further analyzed by EDS mapping. The EDS mapping images of the elements V, O, and S in Figure 2f–h show that all the elements are distributed homogeneously in P-LVO, indicating that PEDOT:PSS was uniformly deposited on P-LVO without severe phase separation. High-resolution TEM images of LVO and P-LVO are shown in Figure 2i,j, respectively, to further monitor the details before and after surface modification. The TEM images of both samples exhibit distinct lattice fringe spacing of 0.39 nm, which matches well with the lattice spacing (011) of orthorhombic LVO [28]. Moreover, compared with the pristine sample, the P-LVO displays a PEDOT:PSS layer with a thickness of around 2–5 nm uniformly coated on its surface. The selected-area electron diffraction (SAED) pattern, as shown in the inset of Figure 2i, also confirms the highly crystal structure, which is in good agreement with the XRD analysis (Figure 1a). It is expected that the PEDOT:PSS layer can improve the electrical and ionic conductivities of the LVO, leading to enhanced rate performance.

**Figure 3:**
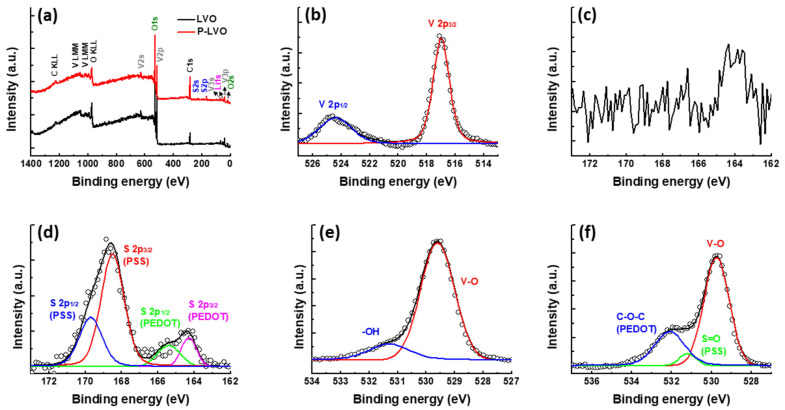
Chemical valence state of LVO and P-LVO. (**a**) XPS survey of the LVO and P-LVO; (**b**) V 2p XPS spectrum of P-LVO; S 2p XPS spectrum of (**c**) LVO and (**d**) P-LVO; and O 1s XPS spectrum of (**e**) LVO and (**f**) P-LVO.

The effect of surface coating on the valence state of LVO was investigated by using XPS measurement. Figure 3a shows the full XPS survey of the LVO and P-LVO. Compared with the pristine sample, the P-LVO not only presents the peaks of Li 1s, V 2p, O 1s, and C 1s but also shows an additional peak of S 2p, indicating the existence of PEDOT:PSS cast on the surface. The high-resolution XPS spectrum of V 2p for the P-LVO is shown in Figure 3b. The V 2p spectrum of the P-LVO comprises two main peaks located at 516.9 and 524.4 eV, which can be assigned to the spin–orbit splitting of the components V^5+^ 2p_3/2_ and V^5+^ 2p_1/2_, respectively [29]. This result suggests that the surface modification with PEDOT:PSS cannot alter the valence state of the LVO. In addition, the high-resolution XPS spectra of S 2p for the two samples are presented in Figure 3c,d. It can be found that the S element is absent in the pristine LVO sample, as shown in Figure 3c. In contrast, the S 2p spectrum of P-LVO (Figure 3d) is consistent with the previous literature [30], which shows that the S 2p XPS peak consists of two main peaks at 164.3 and 168.6 eV. The lower (164.3 eV) and higher (168.6 eV) binding energy peaks belong to the S atoms in the PEDOT polymer chains and the PSS fragments, respectively. The O1s spectrum of pure LVO displayed in Figure 3e shows a broad asymmetrical peak, which can be de-convoluted into two peaks centered at 529.6 and 531.4 eV, respectively [29]. They are attributed to crystal lattice O atoms (V−O) in LVO and a surface hydroxyl group, respectively. In the O 1s spectrum of P-LVO, as shown in Figure 3f, the de-convoluted peaks at 529.7, 531.2, and 532.1 eV are assigned to the V−O, S=O (PSS), and C−O−C (PEDOT) bond, respectively [31,32]. These results further confirm that the PEDOT:PSS was successfully deposited on the LVO surface.

**Figure 4:**
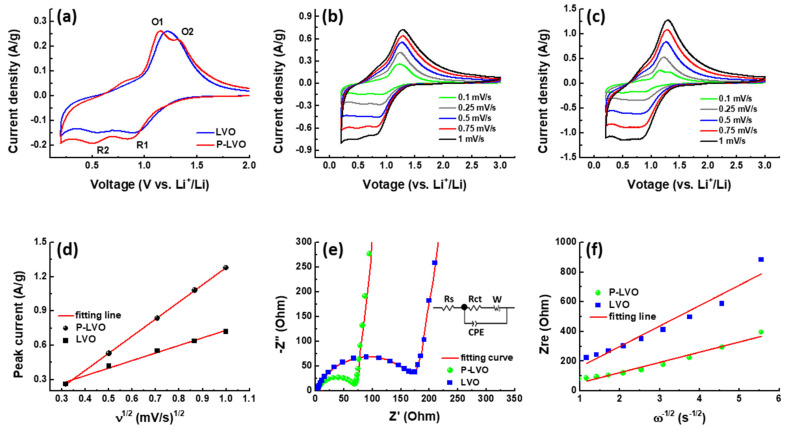
The electrochemical properties for the LVO and P-LVO. (**a**) the comparison of CV curves of LVO and P-LVO stepped at 0.1 mV/s; the CV curves of (**b**) LVO and (**c**) P-LVO at different scan rates; (**d**) linear relationships between the anodic peak current densities (*i*) and the square root of the scan rate (ν^1/2^); (**e**) Nyquist plots of the pristine LVO and P-LVO (inset: equivalent circuit model used to fit the EIS data); and (**f**) plots of real parts of the complex impedance versus ω^−1/2^.

Figure 4a shows the comparison of CV curves for pristine LVO and P-LVO at a scan rate of 0.1 mV/s. There are two redox couples at a voltage of 1.15 V/0.52 V and 1.33 V/0.88 V for the P-LVO, which corresponds to the transition between V^5+^ and V^3+^ due to the lithiation and delithiation processes [33]. Compared with the P-LVO, the CV curve of the LVO electrode exhibits broader and weaker peaks with smaller areas, indicating its lower electrochemical activity. The CV curves of the two electrodes stepped at different scan rates are presented in Figure 4b,c. The oxidation and reduction peaks shift to higher and lower voltage regions with increasing scan rates, suggesting higher polarization under a high scan rate. The peak current (*i*) of the two electrodes exhibits a linear relation with the square root of the scan rate (ν^1/2^), which is considered diffusion-controlled progress rather than surface control, as shown in Figure 4d. From the linear relationship, it can be seen that the P-LVO electrode shows a larger slope than that of LVO, indicating its faster ionic transport based on the Randles-Ševčík equation. To study the effect of PEDOT:PSS modification on the electronic conductivity properties of LVO, the EIS measurements were carried out, as shown in Figure 4e. The EIS curves of the two electrodes are composed of a semicircle and straight line at the high/intermediate and low-frequency regions, respectively. The charge transfer resistance (*R*ct) of LVO and P-LVO obtained by fitting the EIS curves with the equivalent circuit inserted in Figure 3e are found to be 196.1 and 76.6 Ω, respectively. The reduction of *R*ct for P-LVO verifies that the surface-modified LVO has higher conductivity than the pristine one. The plot of Z_re_ versus the reciprocal square root of the angular frequency (ω^−0.5^) is presented in Figure 4f. From the extracted slope of the Z_re_ − ω^−0.5^ plot, we can calculate the diffusion coefficient (*D*_Li_) according to the following equations [34]:

Z_re_ = *R*ct + Rs + σω^−1/2^ (1)

*D*_Li_ = R^2^T^2^/2A^2^*n*^4^F^4^C^2^σ^2^ (2)

where σ, R, T, F, A, and C are Warburg impedance coefficient, gas constant, absolute temperature, Faraday’s constant, area of the electrode surface, and the molar concentration of Li^+^ ions. The calculated *D*_Li_ value of P-LVO is 4.4 × 10^−13^ cm^2^/s, which is higher than that of pristine LVO (1.1 × 10^−13^ cm^2^/s), suggesting that the deposited PEDOT:PSS layer can effectively improve the Li^+^ ion mobility of LVO.

**Figure 5:**
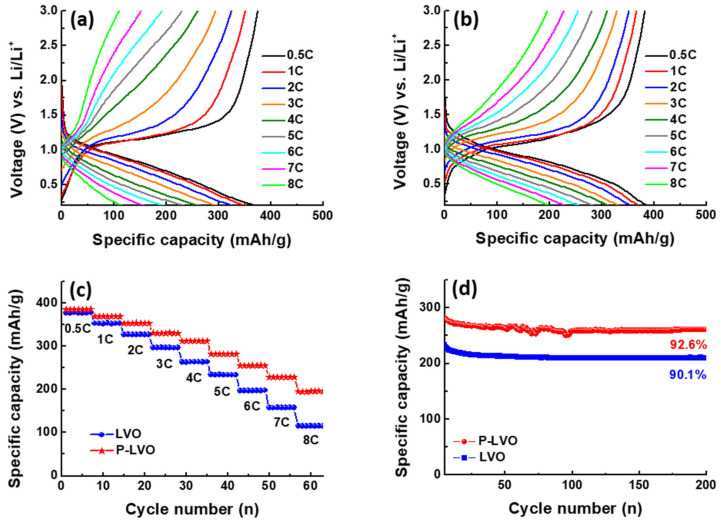
The energy storage performance of the LVO and P-LVO. The GCD profiles of (**a**) pristine LVO and (**b**) P-LVO in the range between 0.2 and 3.0 V (vs. Li^+^/Li) at various current densities from 0.5 to 8 C rate; (**c**) the rate performance of LVO and P-LVO; and (**d**) cycling performance of LVO and P-LVO at 5 C.

Figure 5a,b present the galvanostatic charge–discharge (GCD) curves of LVO and P-LVO at different C rates, with a cut-off voltage set between 0.2 V and 3.0 V vs. Li^+^/Li. At 0.5 C, the pristine LVO electrode delivers a specific capacity of 371.7 mAh/g, while the P-LVO electrode achieves a higher specific capacity of 383.6 mAh/g. However, the capacities of both electrodes decrease with increasing C rate, indicating polarization. Compared to LVO, P-LVO displays lower polarization and higher reaction kinetics. Figure 5c compares the rate performance of the two electrodes. At various current densities, P-LVO delivers higher capacities of 383.6, 365.8, 351.1, 330.6, 308.8, 281.8, 255.2, 224.9, and 191.9 mAh/g, while LVO only delivers capacities of 371.7, 349.1, 325.3, 296.5, 259.8, 232.7, 192.7, 155.1, and 111.3 mAh/g. Additionally, Figure 5d shows the cyclic performances of the two electrodes at 5 C. After 200 cycles, the capacity retentions of LVO and P-LVO electrodes are 90.1% and 92.6%, respectively, indicating superior cycling stability. The PEDOT:PSS coating provides several advantages for the LVO electrode material. Firstly, it offers protection to the LVO particles from the corrosive electrolyte environment during long cycling, which contributes to improving the cycling stability of the electrode. Secondly, the PEDOT:PSS coating provides a more conductive pathway for electrons, resulting in faster Li^+^ ion migration. The balance between electronic and ionic transport is crucial for achieving the high-rate performance of the LVO electrode.

**Figure 6:**
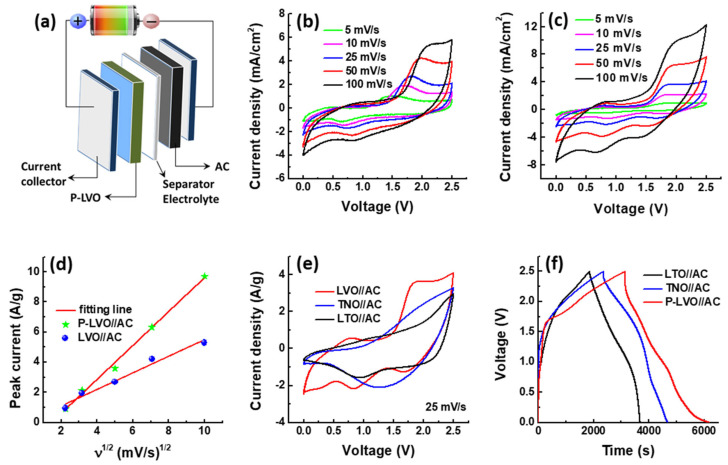
The electrochemical performance of the LIC based on AC and different negative electrodes. (**a**) Schematic illustration of the assembled structure of the P-LVO//AC LIC; the CV curves of (**b**) LVO//AC LIC and (**c**) P-LVO//AC LIC with various scan rates; (**d**) linear relationships between the anodic peak current densities and the square root of the scan rate (ν^1/2^); (**e**) CV curves (25 mV/s); and (**f**) GCD curves of the LICs with different negative electrodes at 0.1 A/g.

The LICs based on P-LVO and AC were fabricated to evaluate the practical application of the P-LVO and the P-LVO//AC structure and are shown in Figure 6a. The CV curves of the LVO//AC and P-LVO//AC devices with different scan rates are compared, as shown in Figure 6b,c. The shape of the CV curves is not severely distorted with increasing scan rates, demonstrating the superior rate capability and good reversibility of the P-LVO//AC LIC. It can be seen that the P-LVO//AC device shows a higher current density with a larger CV area compared with that of the LVO//AC device. In addition, the *i* − ν^1/2^ plot, as shown in Figure 6d, also indicates that P-LVO//AC exhibits a higher slope, suggesting P-LVO//AC possesses greater electrochemical activity and faster kinetics. Based on the superior electrochemical characteristics, in this study, we also compare the energy storage properties of P-LVO//AC with those of LTO//AC and TNO//AC. The CV curves of the three devices recorded at 25 mV/s are presented in Figure 6e. Clearly, the P-LVO//AC LIC shows the largest CV area among the three LICs, implying its largest specific capacitance due to the higher theoretical capacity of LVO (592 mAh/g) than that of LTO (175 mAh/g) and TNO (387.6 mAh/g). Figure 6f shows the GCD curves of the three LICs at the current density of 0.1 A/g. The specific capacitance of the P-LVO//AC device at 0.1 A/g is calculated to be 123.3 F/g, which is much larger than that of the LTO//AC (72.9 F/g) and TNO//AC LICs (93.0 F/g). The XRD patterns of LTO and TNO are provided in the Supporting Information (Figure S1).

**Figure 7:**
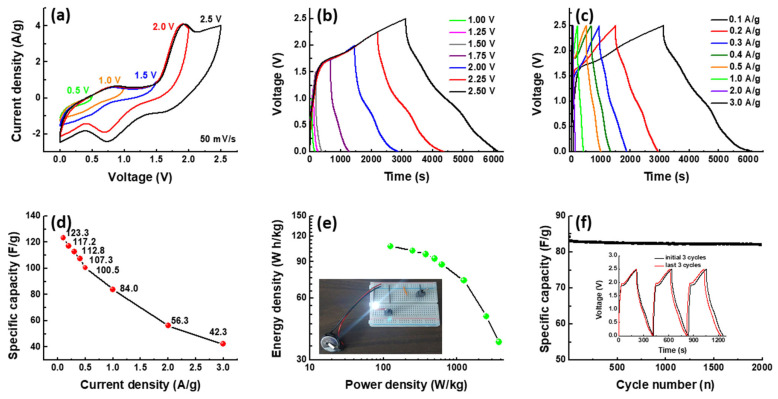
The performance of P-LVO//AC LIC. (**a**) CV curves and (**b**) GCD curves of the P-LVO//AC LIC, recorded in various potential windows; (**c**) GCD curves of the P-LVO//AC LIC at different current densities; (**d**) specific capacitances of P-LVO//AC LIC recorded at various current densities; (**e**) Ragone plots of the P-LVO//AC LIC (inset: digital photograph of an LED lighted by the LIC); and (**f**) cycling performance of the LIC at 1.0 A/g for 2000 cycles (The inset shows the corresponding GCD curves of the initial 3 cycles and the last 3 cycles).

As previously stated, due to its high theoretical capacity, the P-LVO material synthesized in this study is a promising candidate for high-performance LIC electrodes. To further investigate its electrochemical properties, the CV and GCD curves of the P-LVO//AC LIC at various voltage windows at 50 mV/s and 0.1 A/g, respectively, are presented in Figure 7a,b. It is evident from both the CV and GCD curves that the majority of the capacitance is stored within the voltage range of 1.5 to 2.5 V for the P-LVO//AC LIC. The rate capability of the LIC was evaluated by performing GCD tests at various current densities ranging from 0.1 to 3.0 A/g. The GCD curves of the P-LVO//AC LIC at different current densities, as shown in Figure 7c, demonstrate high coulombic efficiency and mild IR drop at each current density. The rate performance of the P-LVO//AC LIC is presented in Figure 7d. The specific capacitance of the LIC, calculated based on the GCD curves shown in Figure 7c, was found to be 123.3 F/g at the current density of 0.1 A/g, retaining 34.3% of the initial value at the current density of 3 A/g. Figure 7e shows the Ragone plot, which illustrates the relationship between the power and energy density of the P-LVO//AC LIC. The LIC exhibits a maximum energy density of 107.0 W h/kg at a power density of 125 W/kg. Even at an ultrahigh power density of 3750 W/kg at 3 A/g, it still maintains an energy density of 36.7 W h/kg. The energy density of the P-LVO//AC LIC is competitive or higher than those of previously reported LICs based on LTO and TNO, such as LTO/C//PGM (72 W h/kg) [35], LTO//NGO (26.15 W h/kg) [36], LTO/P-MWCNT//AC (70.9 W h/kg) [37], P-LTO//AC (85.7 W h/kg) [38], G–LTO//G–SU (95 W h/kg) [39], TNO@MS/C//AC (147.2 W h/kg) [9], TNO-750-7h//AC (100.6 W h/kg) [40], and TNO//graphene (74 W h/kg) [41]. The image of a lighted LED driven by the P-LVO//AC LIC is also shown in the insert of Figure 7e. The cycling life of the P-LVO//AC LIC was also evaluated in the range of 0–2.5 V at 1.0 A/g, as shown in Figure 7f. The LIC exhibits a capacitance retention of 97.4% after 2000 cycles. Moreover, the GCD profiles of the last three cycles are almost identical to those of the initial three cycles (inset in Figure 7f), confirming its excellent electrochemical reversibility and great cycling stability. Based on the above analysis, it can be concluded that the LIC combined with P-LVO and AC can be a qualified candidate for future energy storage devices with excellent energy densities and remarkable cyclability.

## 4. Conclusions

In summary, we prepared PEDOT:PSS modified LVO by dispersing LVO in PEDOT:PSS and removing the solvent via rotary evaporation to form the P-LVO composite. The homogeneously modified PEDOT:PSS layer with a thickness of around 2–5 nm did not alter the crystalline structure or chemical valence state of LVO. The conductive PEDOT:PSS layer reduced the resistance of LVO, leading to improved electrical conductivity and Li^+^ diffusion coefficient. P-LVO exhibited lower *R*ct (76.6 Ω) and larger *D*_Li_ (4.4 × 10^−13^ cm^2^/s) compared to pristine LVO (196.1 Ω and 1.1 × 10^−13^ cm^2^/s, respectively). As a result, P-LVO demonstrated superior electrochemical performance, including remarkable rate capability (191.9 mAh/g at 8 C) and great cycling life (only 7.4% capacity fading after 200 cycles at 5 C). In contrast, pristine LVO could only deliver a capacity of 111.3 mAh/g at 8 C. We also demonstrated LICs composed of P-LVO anode and AC cathode, delivering an energy density of 107.0 Wh/kg at a power density of 125 W/kg with excellent cycling stability. The high-rate P-LVO with a large capacity can be a promising alternative candidate to replace LTO as an anode material in LIB and LIC.

## Data Availability

All data are contained within the article and Supplementary Material are available upon request from the authors.

## Supplementary Materials

The following supporting information can be downloaded at: https://www.mdpi.com/article/10.3390/polym15112502/s1, Figure S1. The XRD patterns of (a) LTO and (b) TNO anode materials.
