# Supplementary material for: Surface Modification of Li3VO4 with PEDOT:PSS Conductive Polymer as an Anode Material for Li-Ion Capacitors
Source: Polymers (Basel). 2023 May 29;15(11):2502. doi: 10.3390/polym15112502 (PMC10255298; doi:10.3390/polym15112502)
Supplement: Supplementary file 1 [file polymers-15-02502-s001.zip › polymers-2366756-supplementary.pdf]

Supporting Information

Surface modification of Li_3VO_4 with PEDOT:PSS conductive polymer as an anode material for Li–ion capacitors

Shih–Chieh Hsu 1, Kuan–Syun Wang 2, Yan–ting Lin 3, Jen–Hsien Huang 4, Nian–Jheng Wu 5, Jia–Lin Kang 6,*, Huei–Chu Weng 7,*, and Ting–Yu Liu 2, 8 *

[1] Department of Chemical and Materials Engineering, Tamkang University, No. 151, Yingzhuan Road, Tamsui District, New Taipei City 25137, Taiwan;
[2] Department of Materials Engineering, Ming Chi University of Technology, 84 Gungjuan Road, Taishan District, New Taipei City 24301, Taiwan;
[3] Institute of Nuclear Energy Research, Atomic Energy Council, 1000 Wenhua Road, Jiaan Village, Longtan District, Taoyuan City 32546, Taiwan;
[4] Department of Green Material Technology, Green Technology Research Institute, CPC Corporation, No.2, Zuonan Rd., Nanzi District, Kaohsiung City 81126, Taiwan;
[5] Université Paris–Saclay, CNRS, Institut des Sciences Moléculaires d'Orsay, 91405 Orsay, France;
[6] Department of Chemical and Materials Engineering, National Yunlin University of Science and Technology, Douliu 64002, Taiwan
[7] Department of Mechanical Engineering, Chung Yuan Christian University, No. 200, Chungpei Road, Chungli District, Taoyuan City 32023, Taiwan
[8] Department of Chemical Engineering and Materials Science, Yuan Ze University, Taoyuan City 32003, Taiwan

1. Electrochemical Characterizations

First, LVO powders, conductive carbon and polyvinylidene fluoride (PVDF) binder were homogeneously mixed in N-methyl pyrrolidinone (NMP) in a weight ratio of 80:15:5 by a 3D mixer (BiO-COMPONENTS™ Inversina 2L) for 4h. Then, the slurry was coated on Al foil and dried at 80°C for 2 h. The electrodes were punched in the form of 14 mm diameter disks. Before the fabrication of coin cell, the punched disks were stored in vacuum and heated at 80°C for 24 h. The coin cells were fabricated by using LVO electrode metallic lithium and polypropylene membrane as working, counter electrodes and separator, respectively. All the assembled process was performed in an argon-filled glove box. The electrolyte comprises 1.0 M $LiPF_6$ dissolved in a mixture of ethylene carbonate and dimethyl carbonate at a volumetric ratio of 1:1. Cyclic voltammogram (CV) was measured with an electrochemical workstation (PAR, PARSTAT 4000A). Electrochemical impedance spectrum (EIS) was carried out at frequencies between 10^5 Hz and 10^{-2} Hz on an Autolab electrochemical workstation.

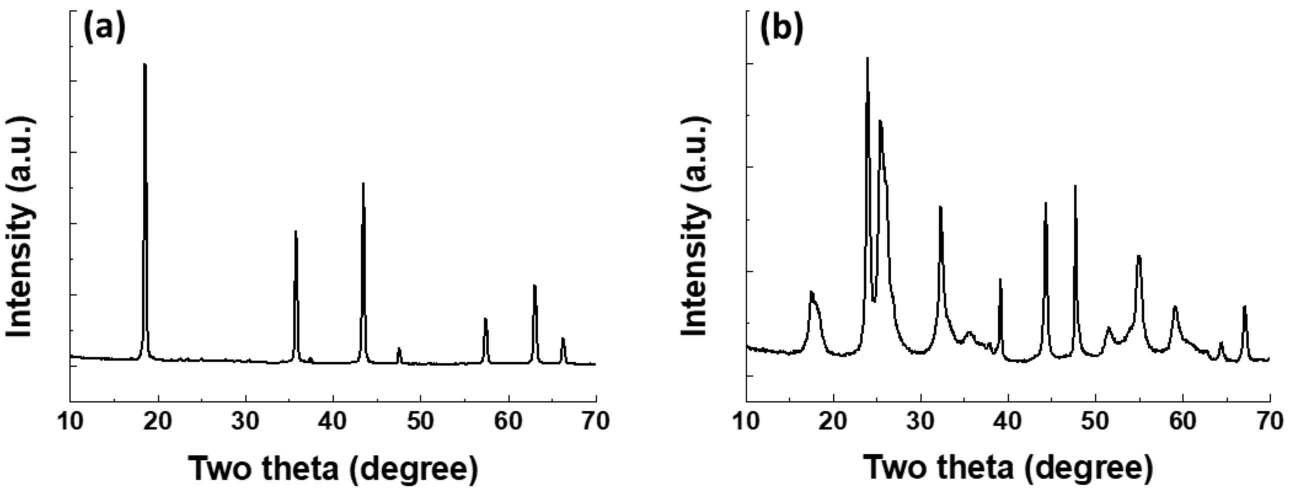

Figure S1. The XRD patterns of (a) LTO and (b) TNO anode materials.
